# TagDigger: user-friendly extraction of read counts from GBS and RAD-seq data

**DOI:** 10.1186/s13029-016-0057-7

**Published:** 2016-07-11

**Authors:** Lindsay V. Clark, Erik J. Sacks

**Affiliations:** Department of Crop Sciences, University of Illinois at Urbana-Champaign, 1201 W. Gregory Drive, Urbana, IL 61802 USA

**Keywords:** Genotyping-by-sequencing, Meta-analysis, Read depth, Restriction site-associated DNA sequencing, Single nucleotide polymorphism (SNP), Tag counts

## Abstract

**Background:**

In genotyping-by-sequencing (GBS) and restriction site-associated DNA sequencing (RAD-seq), read depth is important for assessing the quality of genotype calls and estimating allele dosage in polyploids. However, existing pipelines for GBS and RAD-seq do not provide read counts in formats that are both accurate and easy to access. Additionally, although existing pipelines allow previously-mined SNPs to be genotyped on new samples, they do not allow the user to manually specify a subset of loci to examine. Pipelines that do not use a reference genome assign arbitrary names to SNPs, making meta-analysis across projects difficult.

**Results:**

We created the software TagDigger, which includes three programs for analyzing GBS and RAD-seq data. The first script, *tagdigger_interactive.py*, rapidly extracts read counts and genotypes from FASTQ files using user-supplied sets of barcodes and tags. Input and output is in CSV format so that it can be opened by spreadsheet software. Tag sequences can also be imported from the Stacks, TASSEL-GBSv2, TASSEL-UNEAK, or pyRAD pipelines, and a separate file can be imported listing the names of markers to retain. A second script, *tag_manager.py*, consolidates marker names and sequences across multiple projects. A third script, *barcode_splitter.py*, assists with preparing FASTQ data for deposit in a public archive by splitting FASTQ files by barcode and generating MD5 checksums for the resulting files.

**Conclusions:**

TagDigger is open-source and freely available software written in Python 3. It uses a scalable, rapid search algorithm that can process over 100 million FASTQ reads per hour. TagDigger will run on a laptop with any operating system, does not consume hard drive space with intermediate files, and does not require programming skill to use.

**Electronic supplementary material:**

The online version of this article (doi:10.1186/s13029-016-0057-7) contains supplementary material, which is available to authorized users.

## Background

Genotyping-by-sequencing (GBS), and closely-related techniques such as restriction-site associated DNA sequencing (RAD-seq) and double-digest restriction-site associated DNA sequencing (ddRAD), have revolutionized the use of molecular markers in model and non-model organisms by providing inexpensive methods to simultaneously mine and genotype thousands of single nucleotide polymorphism (SNP) markers without the need for a reference genome [1–4]. However, data quality remains a major issue with all of these techniques [4, 5]. Because the DNA that is sequenced is a random sample of all sites adjacent to a particular restriction enzyme cut site, for any given individual many loci will be missing simply due to under-sampling [1, 6]. Moreover, in an individual that is heterozygous at a given locus, it is possible that only one of two alleles will be sequenced, making the individual erroneously appear homozygous [6, 7]. A crucial piece of information for evaluating GBS data quality is therefore the number of sequencing reads per allele in each individual. For example, an individual with 20 reads for one allele and zero reads for the other is likely to be a true homozygote, whereas an individual with only one read for one allele and zero reads for the other might be an under-sampled heterozygote. Where genotype accuracy is important, it is practical to simply remove all homozygous genotype calls below a certain read depth [8]. Methods also exist that take read depth into account when estimating allele frequencies and probabilities that genotype calls are correct [9] and for estimating relatedness coefficients [10]. On the other hand, tags with unusually high read depth may represent repetitive sequence and should therefore be excluded from analysis [11]. Knowledge of the number of reads per allele is also important for assigning allelic configurations and estimating allele frequencies in polyploids [12, 13].

Given the importance of read depth in evaluating genotype quality and performing downstream analysis of GBS data, one would expect read counts to be accurately exported from all SNP calling pipelines in an easily-accessible format, but this is not the case. TASSEL’s UNEAK pipeline [6] (for species without a reference genome) exports a text file containing read counts for all SNP calls as part of the final output, but does not report read counts higher than 127. This maximum number of reported read counts is not an issue for filtering out genotypes with low read counts, but does interfere with analysis of polyploid species or bulked samples, for which accurate calculation of read count ratios is needed. TASSEL’s GBS version 2 pipeline [14] (for species with a reference genome) exports accurate read counts in VCF format only, requiring moderate programming skill to extract those values for analysis. Stacks [15] exports read counts, although it has the disadvantage of only running on Unix-like operating systems. pyRAD [16] exports genotypes but not read counts. RADtools [17] exports read counts in a custom format, but is no longer being updated since Stacks was determined to have superior genotyping and performance [5]. RADtools also does not allow barcode lengths other than 5 nucleotides, and does not allow multiple barcodes per individual.

For tasks such as genomic selection or the assignment of new individuals to known populations, it is also desirable to call alleles from a user-specified set of SNPs, rather than re-running the entire SNP-mining pipeline. With both TASSEL and Stacks one can run data from new samples against a previously-generated library of SNPs. However, neither program allows the user to specify subset of SNPs to examine. pyRAD and RADtools will only genotype SNPs de-novo; they do not have options to add new samples to an existing genotype set.

For cross-study comparisons, it is also useful to have a universal set of marker names across multiple projects. Reference-genome-based pipelines such as TASSEL-GBS facilitate such comparisons by naming SNPs according to alignment position, but reference-free pipelines assign arbitrary numbers to loci since no other information is available for naming. For pipelines such as TASSEL-UNEAK and Stacks, utilities are needed to compare tag sequences across projects and generate universal sets of marker names.

We present new software, TagDigger, which can manage sets of DNA sequence tags and rapidly search for those tags in FASTQ files. TagDigger is open-source, written in Python 3, and will run on any operating system. It is designed to require minimal RAM and processing power so that it can be run on a laptop computer. It can read FASTQ files in either uncompressed or GZIP format, and does not waste hard drive space by uncompressing zipped files or generating other working files before producing the final output. All input and output files are in comma-separated value (CSV) formats so that they can be created and opened with common software such as R or Microsoft Excel. Tag sequences can additionally be read in the formats output by TASSEL, Stacks, and pyRAD. Lastly, TagDigger is designed to be accessible to users lacking programming experience and avoids the use of incomprehensible error messages.

## Implementation

Currently, three Python programs are included with TagDigger: *tagdigger_interactive.py*, which counts the occurrences of tags in FASTQ files and outputs a table of tag counts indexed by tag and barcode, as well as (optionally) a table of numeric diploid genotypes for biallelic markers; *tag_manager.py*, which consolidates marker names, tag sequences, alignment and other information across multiple projects; and *barcode_splitter.py*, which splits a FASTQ file, by barcode, into multiple FASTQ files with barcode, adapter, and potentially chimeric sequence removed, and optionally generates MD5 checksums for all output files to facilitate archiving data with NCBI (National Center for Biotechnology Information), EBI (European Bioinformatics Institute) or DDBJ (DNA Data Bank of Japan). All functions used by these three programs are contained in the file *tagdigger_fun.py* so that they can be used by Python programmers for tasks such as batch processing. Restriction enzyme cut sites and adapter sequences are also included at the top of the *tagdigger_fun.py* file so that new enzymes and adapters can be easily added.

### Input

For *tagdigger_interactive.py*, a user-generated key file lists all barcodes to search for in each FASTQ file. Three headers are needed for this CSV file: “File” (indicating the name of the FASTQ file), “Barcode” (indicating the barcode sequence), and “Sample”. Other columns are ignored. If FASTQ file names end with “.gz”, they are assumed to be compressed with GZIP, and otherwise they are assumed to be uncompressed. Barcodes that the user does not wish to investigate can be omitted to speed processing time. If the same sample name appears multiple times, tag counts are summed across all instances of that sample. If barcodes have been removed from the FASTQ file, the user can simply list one sample per file and leave the Barcode column blank. A very similar file is needed for *barcode_splitter.py*, but with the headers “Input File”, “Barcode”, and “Output File”.

Tag sequences can be imported to *tagdigger_interactive.py* and *tag_manager.py* in any of seven different formats. Four of these are the direct output of other SNP-calling software: FASTA files from TASSEL-UNEAK, the SAM file used for generating markers in TASSEL-GBS, tab-delimited text output of the *cstacks* program in Stacks, and the*.alleles* file output from pyRAD. Three CSV formats are also readable: two tags per row, with column headers “Marker name”, “Tag sequence 0”, and “Tag sequence 1”; one tag per row (allowing for non-biallelic markers), with column headers “Marker name”, “Allele name”, and “Tag sequence”; and two merged tags per row, with column headers “Marker name” and “Tag sequence”. For the latter format, the variable portion of the tag is put between square brackets with a forward slash separating the two alleles, e.g., ACAGACTT[A/T]GTACCCA. This merged format is also used as the output of *tag_manager.py*, since it conserves hard drive space and RAM and makes it easy for the human eye to see the polymorphism. For any of the seven tag formats, the user can supply, as a separate file, a list of names of markers to include in the output. Supplying such a list conserves processing time and output file size by ignoring tags in which the user is not interested.

### Search algorithm

**Fig. 1:**
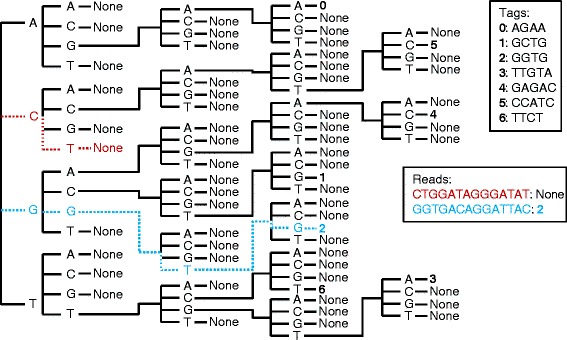
Graphical representation of a sequence indexing tree generated by TagDigger. Use of the tree to match sequencing reads to known tags is illustrated. The red read does not match any known tags, and it takes two steps (looking at the first two nucleotides of the read) to make this determination. The blue read matches one of the expected tags, and it takes four steps to make the match. In comparison, if every read were compared to every tag, seven steps (one for each possible tag) would be required for every read. The maximum number of steps required to match a read will always be the length of the longest tag, which is advantageous when there are thousands of possible tags that are each 40–80 nucleotides long

Before processing a FASTQ file, *tagdigger_interactive.py* recursively builds indexing trees of expected barcode and tag sequences (Fig. 1). These indexing trees enable very rapid matching of sequencing reads to the appropriate barcode and tag. They also enable the software to quickly discard reads that do not match any expected barcode or tag. To save processing time, *tagdigger_interactive.py* ignores sequencing quality scores, with the assumption that a read with an error is unlikely to match a known tag. TASSEL also ignores quality scores, instead distinguishing alleles from errors based on how frequently they appear in the dataset [6, 14]. Because most SNP mining software does not use the entire sequencing read (for example, by default TASSEL uses only the first 64 nucleotides after the barcode, and in other software the reads may be truncated to only retain the highest-quality portion) the TagDigger search algorithm checks to see if the read begins with a barcode and tag, and ignores any nucleotides in the read after the end of the tag. For any given FASTQ read, the algorithm begins at the first nucleotide of the read and moves along the read one nucleotide at a time, navigating through the indexing tree of barcodes. If the read begins with an expected barcode, and also has the expected restriction cut site after the barcode, the algorithm begins navigating the indexing tree of tags, beginning with the first nucleotide in the read after the restriction site (or at the beginning of the restriction site in the case of enzymes such as *Ape*KI with variable cut sites). If the read contains an expected tag, the software increments the read count for that barcode*tag combination.

The *barcode_splitter.py* program uses the above algorithm to assign reads to barcodes. The same search algorithm is used for identifying adapter sequence (to be trimmed out of the read), but the search is performed from the end of the read rather than the beginning. Additionally, the barcode splitter uses more conventional text searching to detect full restriction cut sites present anywhere in the read. Since a full cut site may indicate a chimera of two genomic DNA fragments, sequence after a full cut site is trimmed from the read.

Because the *tag_manager.py* program only assigns markers the same name if tag sequences are an exact match (as opposed to one tag sequence being a truncated version of another), it does not use the same search algorithm as the other two programs. Instead, a binary search using the *bisect* module in Python is used for matching tags across two sets of markers.

### Output

The *tagdigger_interactive.py* program generates a CSV file of read counts, with samples in rows and tags in columns. The first row contains tag names, and the first column contains sample names. Tag names consist of the marker name and allele name(s) separated by an underscore. For biallelic markers, the final allele name is ‘0’ or ‘1’, with the tag that comes first alphabetically being allele ‘0’ (except in formats where the user specifies which tag is ‘0’ and which is ‘1’). The nucleotides at variable sites are also included as an allele name. Because TASSEL-GBS may mine multiple SNPs from the same locus where a tag aligns to the genome, TagDigger does not use the original SNP names when naming tags imported from the TASSEL-GBS pipeline, but can output a table indicating how the TASSEL-GBS SNP names correspond to the marker names generated by TagDigger.

When all markers are biallelic*, tagdigger_interactive.py* can also export a numeric genotype table, with samples in rows and markers in columns. Homozygous genotypes are coded as ‘0’ or ‘2’, heterozygous genotypes are coded as ‘1’, and cells with missing data are left blank. Homozygotes for allele ‘0’ are coded as ‘0’ and homozygotes for allele ‘1’ are coded as ‘2’.

The *barcode_splitter.py* program generates uncompressed FASTQ files. Barcodes, adapter sequence, and potentially chimeric sequence are removed as described in the previous section. The original comment line is retained for each read, with the barcode added to the end of the comment in keeping with Illumina FASTQ format. Optionally, *barcode_splitter.py* can also generate a CSV file listing the MD5 checksum of each new FASTQ file that has been created.

The *tag_manager.py* program outputs a CSV file with the headers “Marker name” and “Tag sequence”. Tag sequences are in the merged format, e.g., CCGATTAG[C/T]AGGGGTT, and can be read back into the TagDigger program. Marker names consist of a prefix supplied by the user followed by a number, e.g., MyLabsTags000102. Optionally, additional columns can contain the original marker names or other data provided by the user in CSV format. A FASTA file of sequences for each marker, with IUPAC nucleotide codes for variable sites, can also be exported. If this FASTA file is used for alignment to a reference genome, *tag_manager.py* can import the resulting SAM file and add columns to the marker list containing chromosome, position, and alignment quality.

## Results and discussion

### Performance

The *tag_manager.py* program was used to generate a consolidated list of tag pairs produced by the UNEAK pipeline across four projects in *Miscanthus sinensis* and *M. sacchariflorus* [8, 18–20]. A total of 57,780 tag pairs were identified, 14,063 of which were shared between at least two projects. When the entire set was imported by *tagdigger_interactive.py*, 5596 out of 57,780 tag pairs were discarded for having an allele in common with another tag pair. The remaining 104,368 tags (52,184 pairs) were then used for evaluating the performance of the search algorithm. Performance was tested on a 2.7 GHz processor using Fedora Linux. For consistency with benchmarking tests performed on other software (see below), 96 Gb of RAM was available to the search algorithm, although it used less than 1 Gb. One thousand randomly sampled sets each of 100, 1000, and 10,000 tags were generated. An indexing tree was built from each set of tags. The time needed to generate indexing trees increased linearly with the number of tags (Table 1). Each tag indexing tree was then used for identifying tags in a FASTQ file, with 96 barcodes, generated in a previous study on *M. sinensis* and *M. sacchariflorus* [18]. For each indexing tree, the time it took to process 10,000 FASTQ reads was recorded. Processing time for the search algorithm increased logarithmically with the number of tags in the indexing tree (Table 1).

**Table 1:** Performance of the TagDigger search algorithm on a FASTQ file from RAD-seq with 96 barcodes

Number of tags	Time to build indexing tree (s)	Time to process 10,000 FASTQ reads (s)	Estimated time to process 200,000,000 FASTQ reads (min)
100	0.03 ± 0.01	0.218 ± 0.016	73
1000	0.84 ± 0.09	0.238 ± 0.005	79
10,000	7.79 ± 1.18	0.291 ± 0.007	97

For each number of tags, 1000 replications were performed with TagDigger, each with a different randomly-sampled subset of tags, and each with a different set of 10,000 reads from the FASTQ file. Means and standard deviations are provided

Performance results indicate that the tag searching algorithm is highly scalable, not unlike a binary search algorithm. For large sets of tags, more processing time (a few seconds) is required to build an indexing tree, but that amount of time remains insignificant compared to the amount of time needed to process an entire FASTQ file (one to two hours for ~200 million reads, the typical output from Illumina HiSeq technology; Table 1). Using our search algorithm, the time to process a FASTQ file is not drastically different whether 100 tags or 10,000 tags are being examined.

Using the same FASTQ file, we benchmarked several popular non-reference de-novo pipelines for processing GBS and RAD-seq data in order to compare them to TagDigger (Table 2). For benchmarking, each pipeline was allowed to use up to 24 2.7 GHz processor cores in parallel, and had access to 96 Gb of RAM. Total processing times were summed across all processor cores in order to compare software, particularly since TagDigger does not use parallel processing. Pipelines tested included UNEAK in TASSEL 3.0, Stacks 1.4 (including *process_radtags* and *denovo_map.pl*), and pyRAD 3.0. Each program was set to recognize *Pst*I and *Msp*I as the restriction sites (for Stacks, only *Pst*I needed to be specified). The minimum number of identical reads needed to create a stack was set to 5 in Stacks in order to match the default in UNEAK. Other parameters were left at defaults. Benchmarking was performed on the same Fedora Linux system on which TagDigger performance was tested.

**Table 2:** Performance of de-novo GBS and RAD-seq pipelines when analyzing a single FASTQ file

Software	Size of intermediate files generated (Gb)	RAM utilized by pipeline (Gb)	Total time, across all processor cores, to process 203,000,000 FASTQ reads and output genotypes (min)
UNEAK pipeline in TASSEL 3.0	0.5	1.9	22
Stacks 1.4	8.2	4.2	424
pyRAD 3.0	40.9	18.5	23,215

The FASTQ file analyzed is the same as that used to produce Table 1. pyRAD differs from UNEAK and Stacks in that it searches for insertions and deletions, whereas the other two only search for substitutions, which is likely to account to for the substantially longer processing time

TASSEL-UNEAK was the only pipeline that was faster than TagDigger, needing twenty minutes to process the file (Table 2), compared to the one to two hours needed by TagDigger (Table 1). However, UNEAK required more RAM and hard drive space than TagDigger. Stacks was the next fastest with seven hours of processing time, and pyRAD was by far the slowest with 387 h of processing time (Table 2), making it impractical for users without access to a computer cluster for parallel processing. UNEAK, Stacks, and pyRAD ranked in the same order in terms of processing time, RAM, and hard drive space needed (Table 2). The main advantage of pyRAD, and the likely reason why it requires long processing times, is its ability to detect insertions and deletions [16]. Given that TagDigger can import the output of pyRAD and genotype the same insertions and deletions on new samples much more quickly and with much less available RAM and hard drive space, we expect TagDigger to be especially useful to pyRAD users. We did not benchmark RADtools on our FASTQ file, given that the file included barcodes of multiple lengths, which is not supported by RADtools.

Since Stacks and pyRAD de-multiplex FASTQ files similarly to *barcode_splitter.py* (producing one FASTQ file per sample, with barcode sequence removed), we also compared processing time across these three programs (Table 3). Stacks and TagDigger had similar processing times, which were approximately half the processing time of pyRAD. Of these three, TagDigger is the only software that can run on Microsoft Windows.

**Table 3:** Performance of software for de-multiplexing a single FASTQ file

Software	Time (min)
*Barcode_splitter.py* in TagDigger	169
Stacks 1.4	156
pyRAD 3.0	358

The FASTQ file analyzed is the same as that used to produce Tables 1 and 2

### User interface

All three of the TagDigger programs can be launched from the operating system’s shell or command prompt. For example, one would use *cd* to navigate into the directory containing the TagDigger programs, then type “python tagdigger_interactive.py” without any additional arguments. The program then prompts the user to supply information such as restriction enzyme, directory for reading and writing files, and names of input and output files. If the user makes a mistake, for example misspelling the name of a restriction enzyme or input file, the software simply prompts them again for that piece of information. Information is printed to the console such as the number of barcodes and tags read from input files. As each FASTQ file is processed, TagDigger also prints its progress to the console so that the user can estimate how much time remains.

## Conclusions

With the increasing popularity of GBS among research groups that lack previous bioinformatics experience, we expect that TagDigger will help many users to more easily manage their data and evaluate genotype quality. Universal marker names generated by TagDigger will make meta-analysis of mapping and association studies more straightforward. Easy accessibility of read count data via TagDigger will facilitate the development of new statistical methodologies that utilize read depth information. Lastly, we hope that TagDigger will encourage the archiving of raw GBS sequence reads in public databases such as NCBI, EBI, and DDBJ by providing a platform-independent tool for splitting FASTQ files by barcode and calculating MD5 checksums. All source code for TagDigger is available with this manuscript (Additional file 1).

## Availability and requirements

**Project name:** TagDigger

**Project home page:**https://github.com/lvclark/tagdigger

**Archived version:** DOI: 10.5281/zenodo.55760

**Operating systems:** Platform independent

**Programming language:** Python

**Other requirements:** Python 3.3 or higher

**License:** GNU GPL v. 3

**Any restrictions to use by non-academics:** none

## Abbreviations

CSV, comma-separated value; DDBJ, DNA Data Bank of Japan; ddRAD, double digest restriction-site associated DNA sequencing; EBI, European Bioinformatics Institute; GBS, genotyping-by-sequencing; IUPAC, International Union of Pure and Applied Chemistry; NCBI, National Center for Biotechnology Information; RAD-seq, restriction site-associated DNA sequencing; SAM, sequence alignment/map; SNP, single nucleotide polymorphism

## Additional file

Additional file 1: Source code for TagDigger version 1.0. (GZ 29 kb)
